# Supplementary figures and images for: Phosphoproteomic Analysis of the Jejunum Tissue Response to Colostrum and Milk Feeding in Dairy Calves during the Passive Immunity Period
Source: Animals (Basel). 2022 Dec 30;13(1):145. doi: 10.3390/ani13010145 (PMC9817995; doi:10.3390/ani13010145)

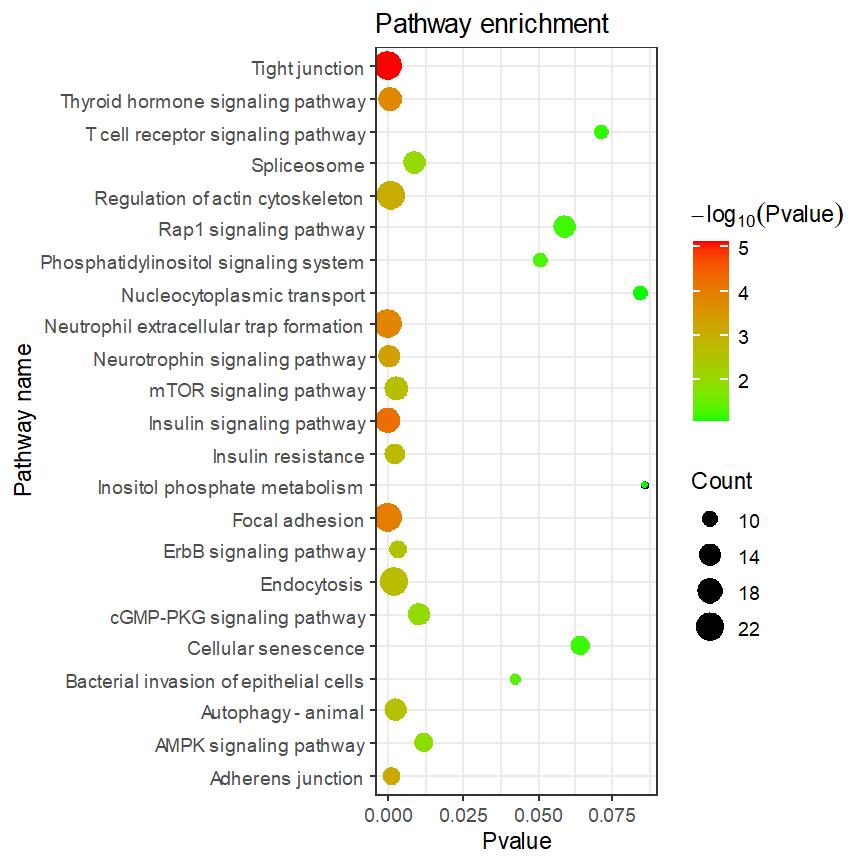

Supplement: Supplementary file 1 [file animals-13-00145-s001.zip › Figure S1.jpg]
